# Supplementary material for: Tracking stimulus representation across a 2-back visual working memory task
Source: R Soc Open Sci. 2020 Aug 5;7(8):190228. doi: 10.1098/rsos.190228 (PMC7481691; doi:10.1098/rsos.190228)
Supplement: Supplementary Figures [file rsos190228supp1.pdf]

Wan, Q., Cai, Y., Samaha, J., & Postle, B.R. (2020). Tracking stimulus representation across a 2-back visual working memory task. *Royal Society Open Science.*

Supplementary Figures

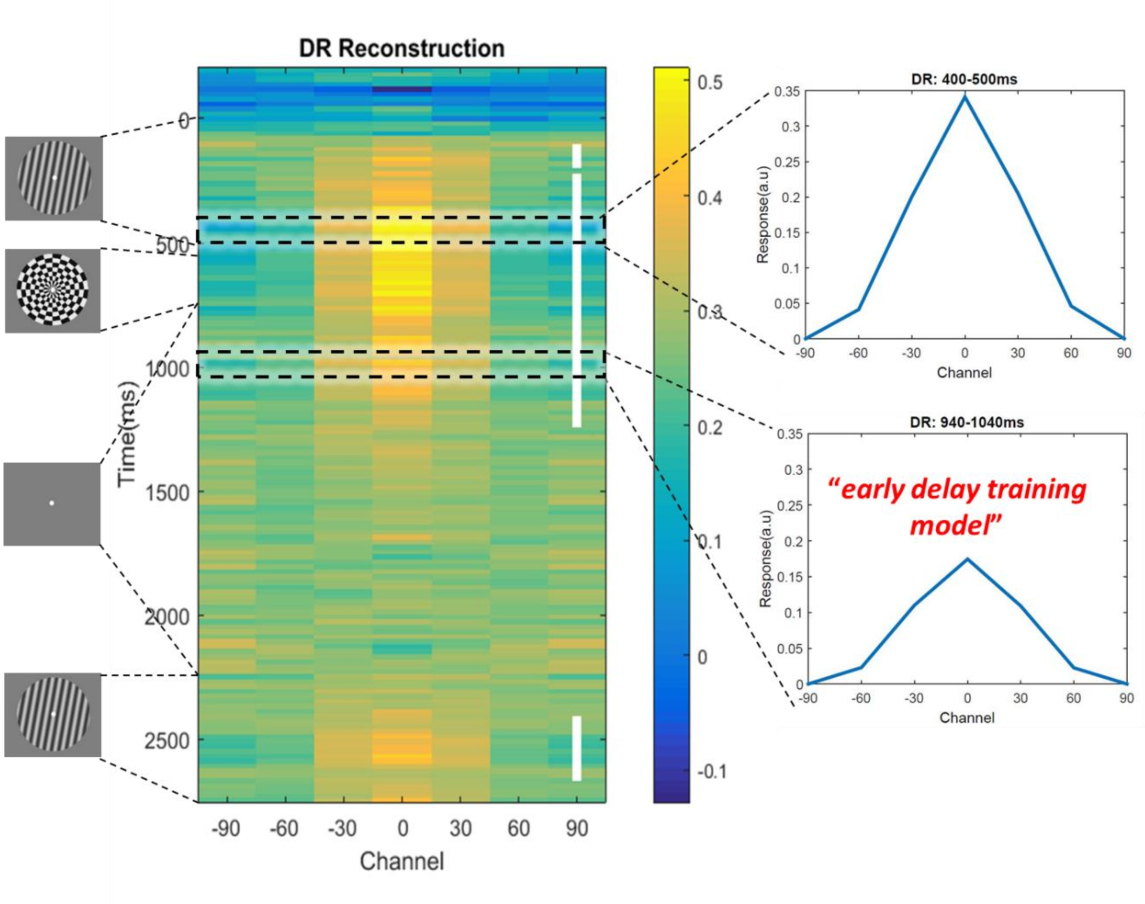

Supplementary Figure 1 (Figure 1 from Stage 1 manuscript). Stimulus reconstruction from the delayed-recognition task in the *Pilot Study*. All conventions are the same as Figure 3.

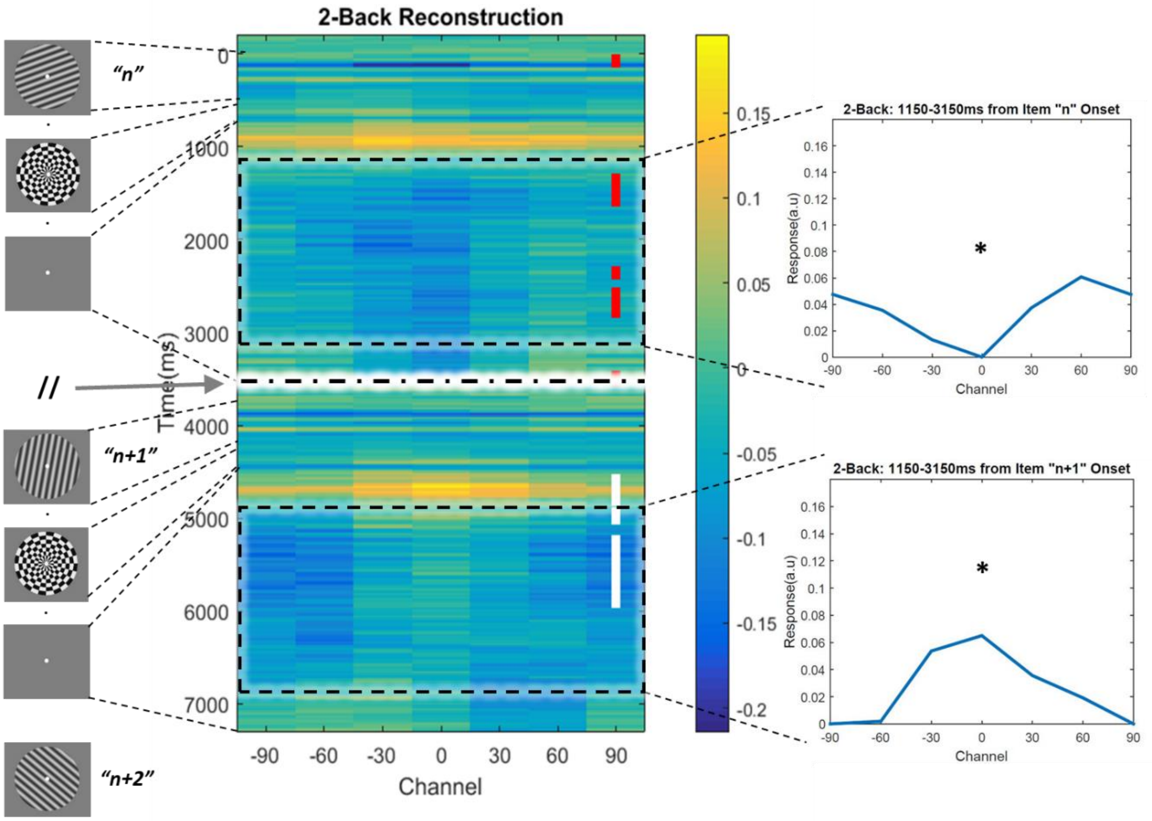

Supplementary Figure 2 (Figure 2 from Stage 1 manuscript). Reconstruction of the 2-back task in the *Pilot Study*. All conventions are the same as Figure 3. (Note that the procedure for cluster-based permutation testing is different, resulting in one change relative to Figure 2 from the Stage 1 manuscript: the loss of significance of one small epoch from each of the ISIs of the 2-back task (3180-3220 ms from item *n* onset and 1330-1370 from item *n + 1* onset).

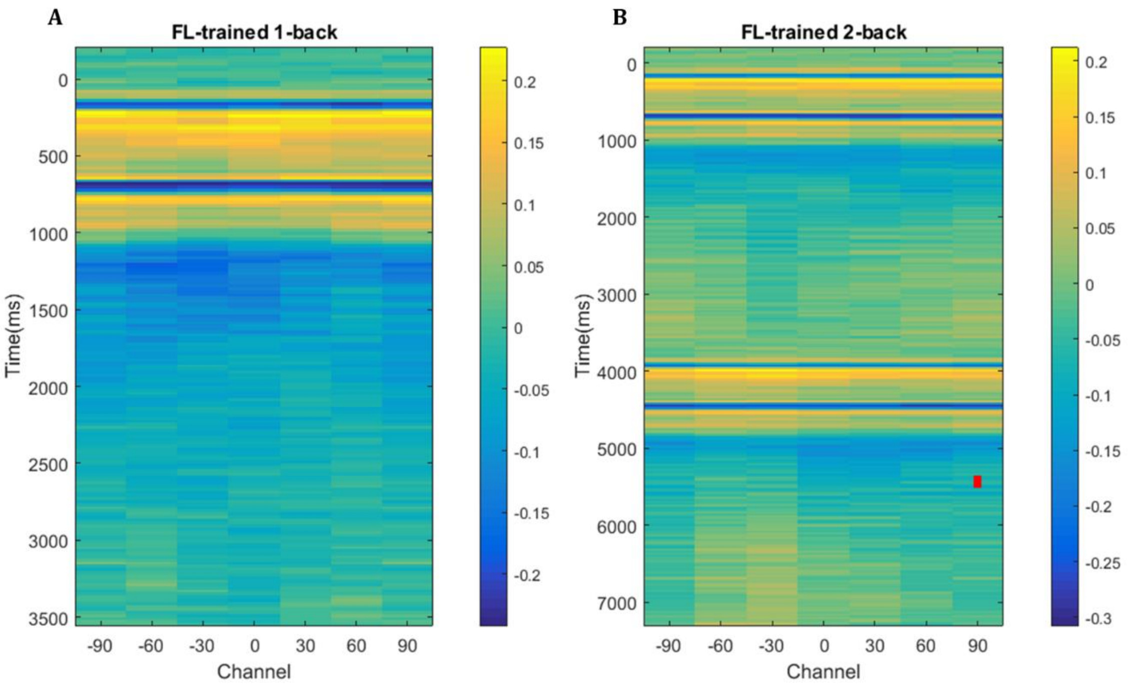

Supplementary Figure 3. Exploratory analyses based on the functional localizer. (A) 1-back and (B) 2-back reconstruction time course based on the functional localizer-trained IEM. All conventions are the same as Figure 3.
